# Supplementary material for: Maternal alcohol consumption and offspring DNA methylation: findings from six general population-based birth cohorts
Source: Epigenomics. 2017 Nov 27;10(1):27–42. doi: 10.2217/epi-2017-0095 (PMC5753623; doi:10.2217/epi-2017-0095)
Supplement: Supplementary file 1 [file epi-10-27-s1.docx]

# File S1

# Cohort-specific information (alphabetical order)

## Cohort-specific methods

**ALSPAC**

Design and study population

ALSPAC is a large, prospective cohort study based in the South West of England. 14,541 pregnant women resident in Avon, UK with expected dates of delivery 1st April 1991 to 31st December 1992 were recruited and detailed information has been collected on these women and their offspring at regular intervals. The study website contains details of all the data that is available through a fully searchable data dictionary (http://www.bris.ac.uk/alspac/researchers/data-access/data-dictionary/).

Written informed consent has been obtained for all ALSPAC participants. Ethical approval for the study was obtained from the ALSPAC Ethics and Law Committee and the Local Research Ethics Committees.

Maternal alcohol consumption

Maternal alcohol consumption during and before pregnancy was self-reported via questionnaire. At a questionnaire sent to participants at around 18 weeks gestation, participants were asked how often they had drunk alcoholic drinks before pregnancy, in the first trimester, and after they had felt the baby move (the second trimester). Options were “never”,”<1glass per week”,”1+ glasses per week”,”1-2 glasses per day”, 3-9 glasses per day”, 10+ glasses per day”. In a questionnaire sent to participants 8 weeks after the birth of the baby, they were asked how often they had drunk alcoholic drinks in the last 2 months of pregnancy. Options were “not at all”, “less than once a week”, “at least once a week”, “1-2 glasses every day”, “at least 3-9 glasses every day”, “at least 10 glasses every day”. These variables were used to define the timing-specific alcohol exposures used in the current study. Binge drinking during any point in pregnancy was defined using information from the questionnaire at 18 weeks plus a questionnaire sent at around 32 weeks gestation. In both questionnaires, participants were asked how many days in the past month they had drunk the equivalent of 2 pints of beer, 4 glasses of wine or 4 pub measures of spirit. Options were “none”, “1-2 days”, “3-4 days”, “5-10 days”, “10+ days”, “everyday”.

Methylation measurements

Cord blood was collected according to standard procedures, spun and frozen at -80˚C. DNA methylation analysis and data pre-processing were performed at the University of Bristol as part of the ARIES project (ariesepigenomics.org.uk). Following extraction, DNA was bisulfite converted using the Zymo EZ DNA MethylationTM kit (Zymo, Irvine, CA). Following conversion, the genome-wide methylation status of over 485,000 CpG sites was measured using the Illumina Infinium® HumanMethylation450k BeadChip assay according to the standard protocol. The arrays were scanned using an Illumina iScan and initial quality review was assessed using GenomeStudio (version 2011.1). The level of methylation is expressed as a “Beta” value (β-value), ranging from 0 (no cytosine methylation) to 1 (complete cytosine methylation). Samples from all time-points in ARIES were distributed across slides using a semi-random approach (sampling criteria were in place to ensure that all time-points were represented on each array) to minimize the possibility of confounding by batch effects. Samples failing quality control (average probe detection p-value ≥ 0.01) were repeated. As an additional quality control step genotype probes on the HumanMethylation450k were compared between samples from the same individual and against SNP-chip data to identify and remove any sample mismatches. Data were pre-processed in R (version 3.0.1) with the WateRmelon package according to the subset quantile normalization approach described by Touleimat & Tost in an attempt to reduce the non-biological differences between probes.

We removed probes that had a detection P-value >0.05 for >5% of samples (3034 probes), probes on the X or Y chromosomes and SNPs (rs probes). 471192 probes remained.

Covariates

Maternal age at delivery was derived from the mother’s report of her own and her baby’s dates of birth. Maternal social class was classified for this study as “attended university” or “did not attend university”. Maternal smoking behaviour was assessed during pregnancy via questionnaire and categorised for this study as 1) never smoking during pregnancy, 2) any smoking during pregnancy. Ten surrogate variables were generated and included in models to adjust for technical batch.

Cell type correction

Cell type correction was applied using the reference-based Houseman method[1] in the minfi package[2]in R[3]. This method estimates the relative proportions of six white blood cell subtypes (CD4+ T-lymphocytes, CD8+ T-lymphocytes, NK (natural killer) cells, B-lymphocytes, monocytes and granulocytes), based on a standard reference population[4].

**GECKO**

Design and study population

The Groningen Expert Center for Kids with Obesity (GECKO) Drenthe cohort is a population-based prospective birth cohort study in Drenthe, a northern province in the Netherlands. All mothers of babies born between April 2006 and April 2007 were invited to participate during the third trimester of pregnancy. Of all 4,778 infants born in this period, a total of 2,874 children (60%) participated in the study and are followed until adulthood. This study has been approved by the Medical Ethical Committee of the University Medical Center Groningen and parents of all participants gave written informed consent. Details about this cohort have been described elsewhere (PMID 18238823).

Maternal alcohol use

Maternal alcohol use before and during pregnancy was self-reported in a questionnaire during the third trimester of pregnancy. The pregnant woman was asked whether she currently consumed alcohol, when answered with “yes” she could give the number of glasses per week. These categories were then reclassified for the current analysis into never, less than 1 glass per week, 1-6 glasses per week, and 7 or more glasses per week. We combined this information with information from two questions whether the woman had consumed alcohol earlier during this pregnancy or before this pregnancy (and the number of glasses per week) and the information given by the midwives about alcohol intoxication (and number of glasses per week) during this pregnancy to define the timing-specific alcohol exposures used in the current study. These categories were then reclassified for the current analysis into never, less than 1 glass per week, 1-6 glasses per week, and 7 or more glasses per week. We classified a woman in the category “sustained drinking” if she consumed alcohol before pregnancy as well as at the time of answering the questionnaire (third trimester) and as “no” if she consumed alcohol before pregnancy, but not during the third trimester. Binge drinking at any point in pregnancy was defined as 5 or more alcoholic consumptions at any time during pregnancy using information from the questionnaire plus the information about alcohol intoxication registered by midwives.

Covariates

Data on maternal age, educational level and smoking were self-reported in questionnaires during the third trimester of pregnancy. Maternal age was used as a continuous covariate. Maternal education was categorized into lower (less than university) and higher (university). Maternal smoking was defined as no vs any smoking during pregnancy. Analyses were additionally adjusted for batch effects by adding plate number as a covariate.

Methylation measurements

Within the GECKO Drenthe birth cohort we selected 258 infants for the methylation study: 129 exposed to maternal smoking during pregnancy and 129 unexposed to both maternal and paternal smoking during pregnancy. From these 258 infants, we used DNA which was extracted from cord blood for the epigenome-wide DNA methylation analyses. To limit batch effects, we randomized all samples over the 96-well plates, based on gender and smoking status. Samples (500 ng per sample) were placed on three 96-well plates. Bisulfite conversion was performed using the EZ-96 DNA methylation kit (Zymo research Corporation, Irvine, USA). Then we used the Infinium HumanMethylation450 BeadChip (Illumina Inc., San Diego, USA) to measure the methylation level as a beta value ranging from zero (no methylation) to one (complete methylation). During the quality control, we excluded two males that clustered in the female group, based on X chromosome betas, which was probably due to maternal blood contamination. We performed Illumina-suggested background normalization, colour correction and Subset-quantile Within Array Normalization (SWAN). We excluded one sample because it did not meet the criteria of ≥99% of the CpGs with detection p value <0.05. This resulted in 129 exposed and 126 unexposed children. We excluded control probes, probes on X or Y chromosomes and probes that did not meet our criteria of a detection p value of <0.05 in ≥99% of the samples, resulting in 465,891 remaining CpGs.

Cell type correction

We used the Reinius-based Houseman method [1,4] with the *estimateCellCounts* function in the Minfi package [2] in R [3] to estimate relative proportions of six white blood cell subtypes (CD4+ T-lymphocytes, CD8+ T-lymphocytes, NK (natural killer) cells, B-lymphocytes, monocytes and granulocytes).

**Generation R (GENR)**

Design and study population

The Generation R Study is a population-based prospective cohort study from fetal life onwards in Rotterdam, the Netherlands, which has been described in detail elsewhere.[5] All included children were born between April 2002 and January 2006 and form a largely prenatally enrolled birth cohort that is being followed-up. A total of 9,778 mothers were included, most during pregnancy (response rate at birth 61%). The study has been approved by Medical Ethical Committee of Erasmus MC, University Medical Center Rotterdam and written consent was obtained for all participants.

Maternal alcohol use

Maternal alcohol use before and during pregnancy was assessed by questionnaires in early (<18 weeks gestational age), mid (18-25 weeks gestational age) and late (>25 weeks gestational age) pregnancy. Pregnant women were asked whether they had used any alcoholic drinks before pregnancy, in the first three months of pregnancy and, in the questionnaires in mid and late pregnancy, whether they drank any alcohol in the past two months. If pregnant women had consumed alcohol, they were asked how much (less than 1 unit per week, 1-3 units per week, 4-6 units per week, 1 unit per day, 1-3 units per day, more than 3 units per day). These categories were then reclassified for the current analysis into never, less than 1 unit per week, 1-6 units per week, and 7 or more units per week. Alcohol use during the second and third trimester reflects the average amount across these two trimesters. Sustained drinking was classified as “yes” if the women consumed alcohol before pregnancy as well as during the second or third trimester and as “no” if the women consumed alcohol before pregnancy, but not during the second and third trimester. Women who were not in one of these categories were excluded from the “sustained drinking” analysis. Binge drinking was defined as “yes” if women consumed 6 or more units per occasion at least once during pregnancy and as “no” if women consumed alcohol before pregnancy and consumed alcohol in moderation (no binge drinking) during pregnancy. Women who were not in one of these categories were excluded from the analysis of binge drinking.

Covariates

Information on maternal age, maternal education, and maternal smoking status was collected by questionnaire at enrollment. Maternal age was used as a continuous covariate. Maternal education was categorized into lower (none, primary or secondary education) and higher (more than secondary education). Maternal smoking was classified as no smoking or any smoking during pregnancy. Analyses were additionally adjusted for batch effects by adding plate number as a covariate.

Methylation measurements

Epigenome-wide DNA methylation was measured in 979 Caucasian children, using DNA extracted from cord blood. 500 ng DNA per sample underwent bisulfite conversion using the EZ-96 DNA Methylation kit (Shallow) (Zymo Research Corporation, Irvine, USA). Samples were plated onto 96-well plates in no specific order. Samples were processed with the Illumina Infinium HumanMethylation450 BeadChip (Illumina Inc., San Diego, USA).

Quality control of analyzed samples was performed using standardized criteria. Samples were excluded in case of sample call rate <99%, colour balance >3, low staining efficiency, poor extension efficiency, poor hybridization performance, low stripping efficiency after extension and poor bisulfite conversion, leading to exclusion of 7 samples (6 for low sample call rates, 1 for poor bisulfite conversion). In addition, 2 samples were excluded because of a gender mismatch and 1 sample because of a retracted informed consent, leaving a total of 969 samples in the analysis.

Probes with a single nucleotide polymorphism in the single base extension site with a frequency of > 1% in the GoNLv4 reference panel were excluded[6], as were probes with non-optimal binding (non-mapping or mapping multiple times to either the normal or the bisulphite-converted genome[7], resulting in the exclusion of 49,564 probes, leaving a total of 436,013 probes in the analysis.

We ran DASES normalization using a pipeline adapted from that developed by Touleimat and Tost[8]. DASES normalization includes background adjustment, between-array normalization applied to type I and type II probes separately, and dye bias correction applied to type I and type II probes separately and is based on the DASEN method described by Pidsley et al, but adds the dye bias correction, which is not included in DASEN[9].

Methylation beta values outside the range of (25^th^ percentile - 3*interquartile range) to (75^th^ percentile + 3*interquartile range) were excluded for each CpG.

For this analysis, we used singleton live births with epigenome-wide association arrays and information on maternal alcohol use and complete covariates, giving total sample sizes of 838 for prepregnancy alcohol use, 866 for alcohol use in the first trimester, 851 for alcohol use in the second or third trimester, 723 for sustained alcohol use and 579 for binge drinking.

Cell type correction

Cell type correction was applied using the reference-based Houseman method[1] in the minfi package[2]in R[3]. This method estimates the relative proportions of six white blood cell subtypes (CD4+ T-lymphocytes, CD8+ T-lymphocytes, NK (natural killer) cells, B-lymphocytes, monocytes and granulocytes), based on a standard reference population[4].

**MoBa1 and 2**

Materials and Methods

Participants represent two subsets of mother-offspring pairs from the national Norwegian Mother and Child Cohort Study (MoBa) [10–12]. The years of birth for MoBa participants ranged from 1999-2009. MoBa mothers provided written informed consent. Each subset is referred to here as MoBa1 and MoBa2. MoBa1 is a subset of a larger study within MoBa that included a cohort random sample and cases of asthma at age three years[13]. We previously reported an association between maternal smoking during pregnancy and differential DNA methylation in MoBa1 newborns[14]. We subsequently measured DNA methylation in additional newborns (MoBa2) in the same laboratory (Illumina, San Diego, CA)[15]. MoBa2 included cohort random sample plus cases of asthma at age seven years and nonasthmatic controls. Years of birth were 2002-2004 for children in MoBa1 and 2000-2005 for MoBa2. Both studies were approved by the Regional Committee for Ethics in Medical Research, Norway and the Institutional Review Board of the National Institute of Environmental Health Sciences, USA.

Maternal BMI

Maternal pre-pregnancy BMI was assessed by maternal self-report of pre-pregnancy height and weight in MoBa questionnaire one distributed to all participating women around gestational week 17 for all three datasets.

Covariates

Information on maternal age, parity, maternal education and smoking was collected via questionnaires completed by the mother or from birth registry records as previously described (4). Maternal age was included as a continuous variable. Parity was categorized as 0, or ≥ 1 births. Maternal educational level was categorized into four groups based on years of education: less than high school/secondary school, high school/secondary school completion, some college or university, or 4 years of college/university or more. Maternal smoking status during pregnancy was classified into three groups: non-smoker, stopped smoking in early pregnancy, and smoked throughout pregnancy.

Methylation measurements

Details of the DNA methylation measurements and quality control for the MoBa1 participants were previously described [14] and the same protocol was implemented for the MoBa2 participants. Briefly, umbilical cord blood samples were collected and frozen at birth at -80**°**C. All biological material was obtained from the Biobank of the MoBa study [11]. Bisulfite conversion was performed using the EZ-96 DNA Methylation kit (Zymo Research Corporation, Irvine, CA) and DNA methylation was measured at 485577 CpGs in cord blood using Illumina’s Infinium HumanMethylation450 BeadChip[16] . Raw intensity (.idat) files were handled in R using the *minfi* package19 to calculate the methylation level at each CpG as the beta-value (β=intensity of the methylated allele (M)/(intensity of the unmethylated allele (U) + intensity of the methylated allele (M) + 100)) and the data was exported for quality control and processing. Probe and sample-specific quality control was performed in the MoBa1 and MoBa2. Similar protocols were applied to MoBa1 and Moba2, as follows: Control probes (N=65) and probes on X (N=11 230) and Y (N=416) chromosomes were excluded in both datasets. Remaining CpGs missing > 10% of methylation data were also removed (N=20 in MoBa1, none in MoBa2). Samples indicated by Illumina to have failed or have an average detection p value across all probes < 0.05 (N=49 MoBa1, N=35 MoBa2) and samples with gender mismatch (N=13 MoBa1, N=8 MoBa2) were also removed. We accounted for the two different probe designs by applying the intra-array normalization strategy Beta Mixture Quantile dilation (BMIQ)[17]. The Empirical Bayes method via *ComBat* was applied separately in each dataset for batch correction using the *sva* package in *R* [18]*.*After quality control exclusions , the total sample sizes were 1,068 for MoBa1 and 685 for MoBa2.

Cell type correction

Cell type correction was applied using the reference-based Houseman method[1] in the minfi package[2]in R[3]. This method estimates the relative proportions of six white blood cell subtypes (CD4+ T-lymphocytes, CD8+ T-lymphocytes, NK (natural killer) cells, B-lymphocytes, monocytes and granulocytes), based on a standard reference population[4].

**Project Viva**

Design and study population

Project Viva is a prospective pre-birth cohort of mothers and their children recruited from a multispecialty group practice in Eastern Massachusetts, USA, which has been described in detail elsewhere[20]. The Institutional Review Board of Harvard Pilgrim Health Care approved the study and participating women provided written informed consent. Eligibility requirements were: ability to answer questions in English, at <22 weeks of gestation at study entry, and a singleton pregnancy. Women were enrolled from 1999 to 2002 and enrollment included a total of 2128 live births. Follow up of the children through adolescence is ongoing.

Cord blood DNA methylation assays were completed in 2014 for 507 Viva infants with genetic consent of which 485 passed quality control procedures. The current maternal alcohol consumption analyses were restricted to 311 mothers who reported as non-Hispanic White and were not missing exposure data.

Maternal alcohol consumption

We obtained data on alcohol consumption from a semi-quantitative food frequency questionnaire (FFQ) that expectant mothers completed after the first research visit (median gestational age 9.9 weeks).

Participants endorsed categories of frequency of beverage consumption from “never/less than 1 per month” to a maximum of “4 or more cans/glasses per day”. The time referent was “in the 3 months before you learned you were pregnant”. We defined alcohol consumption “before pregnancy” as the sum of beer, light beer, liquor, white wine, and red wine.

Methylation measurements

Trained medical personnel obtained venous umbilical cord blood samples immediately after delivery, which they promptly stored in a dedicated refrigerator (4ºC) and transported for processing within 24 hours. Trained laboratory staff processed the samples on the same day, and extracted DNA by using the Qiagen Puregene Kit (Valencia, CA). Aliquots were then stored at -80ºC until analysis.

DNA samples were arranged using a stratified randomization to ensure balance of cohort characteristics across sample plates/batches. Samples were bisulfite converted using the EZ-96 DNA Methylation kit (Zymo Research Corporation, Irvine, USA). Illumina FastTrack Microarray Services (San Diego, CA) performed the analyses using the Illumina Infinium HumanMethylation450 BeadChip (Illumina Inc., San Diego, USA). Failing samples were rerun and passing arrays were defined as having >99% of probes with a detection p value <0.05. Samples with identity concerns (inconsistent genotyping and/or inferred sex) were excluded. Standard sample preprocessing included the exclusion of allosomal probes, non-CpG probes, and failing probes (<99% of samples with detection p values <0.05). Further pre-processing and normalization steps included background adjustment via the normal-exponential out-of-band (“noob”) background correction method with dye-bias equalization[21], and further within-array type II probe adjustment using the Beta-Mixture Quantile Dilation (BMIQ) approach[17].

Covariates

The ComBat method was used to adjust the methylation data for sample plate, to reduce potential for bias due to batch effects. Robust regression analyses were then run, adjusting for continuous maternal age (reported at enrolment), smoking status (dichotomous variable due to small Ns; smoked during pregnancy vs never/former), educational status (college graduate vs. not a college graduate) and parity (categorized as 0 [nulliparous] vs. 1 or more [multiparous]).

Cell type correction

Cell type correction was applied using the reference-based Houseman method[1] in the minfi package[2]in R[3]. This method estimates the relative proportions of six white blood cell subtypes (CD4+ T-lymphocytes, CD8+ T-lymphocytes, NK (natural killer) cells, B-lymphocytes, monocytes and granulocytes), based on a standard reference population[4].

References

1. Houseman EA, Accomando WP, Koestler DC, Christensen BC, Marsit CJ, Nelson HH, et al. DNA methylation arrays as surrogate measures of cell mixture distribution. BMC Bioinformatics. 2012;13:86.

2. Aryee MJ, Jaffe AE, Corrada-Bravo H, Ladd-Acosta C, Feinberg AP, Hansen KD, et al. Minfi: a flexible and comprehensive Bioconductor package for the analysis of Infinium DNA methylation microarrays. Bioinformatics. 2014;30:1363–9.

3. R Core Team, R Development Core Team. R: A language and environment for statistical computing. Vienna, Austria: R Foundation for Statistical Computing; 2012.

4. Reinius LE, Acevedo N, Joerink M, Pershagen G, Dahlén SE, Greco D, et al. Differential DNA methylation in purified human blood cells: Implications for cell lineage and studies on disease susceptibility. PLoS One. 2012;7.

5. Kooijman MN, Kruithof CJ, van Duijn CM, Duijts L, Franco OH, van IJzendoorn MH, et al. The Generation R Study: design and cohort update 2017. Eur. J. Epidemiol. 2016;31:1243–64.

6. Genome of the Netherlands Consortium. Whole-genome sequence variation, population structure and demographic history of the Dutch population. Nat. Genet. 2014;46:818–25.

7. Bonder MJ, Kasela S, Kals M, Tamm R, Lokk K, Barragan I, et al. Genetic and epigenetic regulation of gene expression in fetal and adult human livers. BMC Genomics. 2014;15:860.

8. Touleimat N, Tost J. Complete pipeline for Infinium(®) Human Methylation 450K BeadChip data processing using subset quantile normalization for accurate DNA methylation estimation. Epigenomics. Future Medicine Ltd London, UK; 2012;4:325–41.

9. Pidsley R, Y Wong CC, Volta M, Lunnon K, Mill J, Schalkwyk LC. A data-driven approach to preprocessing Illumina 450K methylation array data. BMC Genomics. 2013;14:293.

10. Magnus P, Birke C, Vejrup K, Haugan A, Alsaker E, Daltveit AK, et al. Cohort Profile Update: The Norwegian Mother and Child Cohort Study (MoBa). Int. J. Epidemiol. 2016;45:382–8.

11. Rønningen KS, Paltiel L, Meltzer HM, Nordhagen R, Lie KK, Hovengen R, et al. The biobank of the Norwegian Mother and Child Cohort Study: a resource for the next 100 years. Eur. J. Epidemiol. 2006;21:619–25.

12. Magnus P, Irgens LM, Haug K, Nystad W, Skjaerven R, Stoltenberg C, et al. Cohort profile: the Norwegian Mother and Child Cohort Study (MoBa). Int. J. Epidemiol. 2006;35:1146–50.

13. Håberg SE, London SJ, Nafstad P, Nilsen RM, Ueland PM, Vollset SE, et al. Maternal folate levels in pregnancy and asthma in children at age 3 years. J. Allergy Clin. Immunol. 2011;127:262–4, 264.e1.

14. Joubert BR, Håberg SE, Nilsen RM, Wang X, Vollset SE, Murphy SK, et al. 450K epigenome-wide scan identifies differential DNA methylation in newborns related to maternal smoking during pregnancy. Environ. Health Perspect. 2012;120:1425–31.

15. Joubert BR, Felix JF, Yousefi P, Bakulski KM, Just AC, Breton C, et al. DNA Methylation in Newborns and Maternal Smoking in Pregnancy: Genome-wide Consortium Meta-analysis. Am. J. Hum. Genet. 2016;

16. Bibikova M, Barnes B, Tsan C, Ho V, Klotzle B, Le JM, et al. High density DNA methylation array with single CpG site resolution. Genomics. 2011;98:288–95.

17. Teschendorff AE, Marabita F, Lechner M, Bartlett T, Tegner J, Gomez-Cabrero D, et al. A beta-mixture quantile normalization method for correcting probe design bias in Illumina Infinium 450 k DNA methylation data. Bioinformatics. 2013;29:189–96.

18. Johnson WE, Li C, Rabinovic A. Adjusting batch effects in microarray expression data using empirical Bayes methods. Biostatistics. 2007;8:118–27.

19. Leek JT, Johnson WE, Parker HS, Jaffe AE, Storey JD. The sva package for removing batch effects and other unwanted variation in high-throughput experiments. Bioinformatics. 2012;28:882–3.

20. Oken E, Baccarelli AA, Gold DR, Kleinman KP, Litonjua AA, De Meo D, et al. Cohort profile: project viva. Int. J. Epidemiol. 2015;44:37–48.

21. Triche TJ, Weisenberger DJ, Van Den Berg D, Laird PW, Siegmund KD. Low-level processing of Illumina Infinium DNA Methylation BeadArrays. Nucleic Acids Res. 2013;41:e90.

## Supplemental Acknowledgements

**ALSPAC:** We are extremely grateful to all the families who took part in this study, the midwives for their help in recruiting them, and the whole ALSPAC team, which includes interviewers, computer and laboratory technicians, clerical workers, research scientists, volunteers, managers, receptionists, and nurses. We would like to acknowledge Tom Gaunt, Oliver Lyttleton, Sue Ring, Nabila Kazmi, and Geoff Woodward for their earlier contribution to the generation of ARIES data (ALSPAC methylation data).

**GECKO:** We are grateful to the families who took part in the GECKO Drenthe study, the midwives, gyneacologists, nurses and GPs for their help for recruitment and measurement of participants, and the whole team from the GECKO Drenthe study.

**GENR:** The Generation R Study is conducted by the Erasmus MC, University Medical Center Rotterdam in close collaboration with the School of Law and Faculty of Social Sciences of the Erasmus University Rotterdam, the Municipal Health Service Rotterdam area, Rotterdam, the Rotterdam Homecare Foundation, Rotterdam and the Stichting Trombosedienst & Artsenlaboratorium Rijnmond (STAR-MDC), Rotterdam. We gratefully acknowledge the contribution of children and parents, general practitioners, hospitals, midwives and pharmacies in Rotterdam. The study protocol was approved by the Medical Ethical Committee of Erasmus MC, Rotterdam. Written informed consent was obtained for all participants. The generation and management of the Illumina 450K methylation array data (EWAS data) for the Generation R Study was executed by the Human Genotyping Facility of the Genetic Laboratory of the Department of Internal Medicine, Erasmus MC, the Netherlands. We thank Ms. Sarah Higgins, Ms. Mila Jhamai, Dr. Marjolein Peters, Dr. Lisette Stolk, Mr. Michael Verbiest, and Mr. Marijn Verkerk for their help in creating the EWAS database and the analysis pipeline.

**MOBA (1,2):** We are grateful to all the participating families in Norway who take part in this on-going cohort study.

**Project Viva:** We are indebted to the Project Viva mothers, children and families

## Funding Support

**ALSPAC:** The UK Medical Research Council and the Wellcome Trust (Grant ref: 102215/2/13/2) and the University of Bristol provide core support for ALSPAC. The Accessible Resource for Integrated Epigenomics Studies (ARIES) which generated large scale methylation data was funded by the UK Biotechnology and Biological Sciences Research Council (BB/I025751/1 and BB/I025263/1). Additional epigenetic profiling on the ALSPAC cohort was supported by the UK Medical Research Council Integrative Epidemiology Unit and the University of Bristol (MC_UU_12013_1, MC_UU_12013_2, MC_UU_12013_5 and MC_UU_12013_8), the Wellcome Trust (WT088806) and the United States National Institute of Diabetes and Digestive and Kidney Diseases (R01 DK10324). The funders had no role in study design, data collection and analysis, decision to publish, or preparation of the manuscript.

**GECKO:** The GECKO Drenthe birth cohort was funded by an unrestricted grant of Hutchison Whampoa Ld, Hong Kong and supported by the University of Groningen, Well Baby Clinic Foundation Icare, Noordlease and Youth Health Care Drenthe. This methylation project in the GECKO Drenthe cohort was supported by the Biobanking and Biomolecular Research Infrastructure Netherlands (CP2011-19).

**GENR:** The general design of the Generation R Study is made possible by financial support from the Erasmus Medical Center, Rotterdam, the Erasmus University Rotterdam, the Netherlands Organization for Health Research and Development and the Ministry of Health, Welfare and Sport. The EWAS data was funded by a grant to VWJ from the Netherlands Genomics Initiative (NGI)/Netherlands Organisation for Scientific Research (NWO) Netherlands Consortium for Healthy Aging (NCHA; project nr. 050-060-810) and by funds from the Genetic Laboratory of the Department of Internal Medicine, Erasmus MC. V.W.J. received a grant from the Netherlands Organization for Health Research and Development (VIDI 016.136.361) and a Consolidator Grant from the European Research Council (ERC-2014-CoG-648916). J.F.F. has received funding from the European Union’s Horizon 2020 research and innovation programme under grant agreement No 633595 (DynaHEALTH). This study received funding from the European Union’s Horizon 2020 research and innovation programme (733206, LIFECYCLE). L.D. received funding from the co-funded programme ERA-Net on Biomarkers for Nutrition and Health (ERA HDHL) (ALPHABET project, Horizon 2020 (grant agreement no 696295; 2017), ZonMW The Netherlands (no 529051014; 2017)).

**MOBA (1,2):** The Norwegian Mother and Child Cohort Study are supported by the Norwegian Ministry of Health and Care Services and the Ministry of Education and Research, NIH/NIEHS (contract no N01-ES-75558), NIH/NINDS (grant no.1 UO1 NS 047537-01 and grant no.2 UO1 NS 047537-06A1). MoBa 1 and 2 were supported by the Intramural Research Program of the NIH, National Institute of Environmental Health Sciences (Z01-ES-49019) and the Norwegian Research Council/BIOBANK (grant no 221097).

**Project Viva:** The Project Viva cohort is funded by NIH grants R01 HL111108, R01 NR013945, and R01 HD034568.
