# Supplementary material for: Maternal alcohol consumption and offspring DNA methylation: findings from six general population-based birth cohorts
Source: Epigenomics. 2017 Nov 27;10(1):27–42. doi: 10.2217/epi-2017-0095 (PMC5753623; doi:10.2217/epi-2017-0095)
Supplement: Supplementary file 3 [file epi-10-27-s3.pdf]

File S2

Supplementary Figures

**Forest plots** for the sustained model (without adjustment for cell counts)	Page 2 to 4
**Leave-one-out plots** showing the impact on effect estimates after leaving each cohort out of the meta-analysis	Page 5 to 7

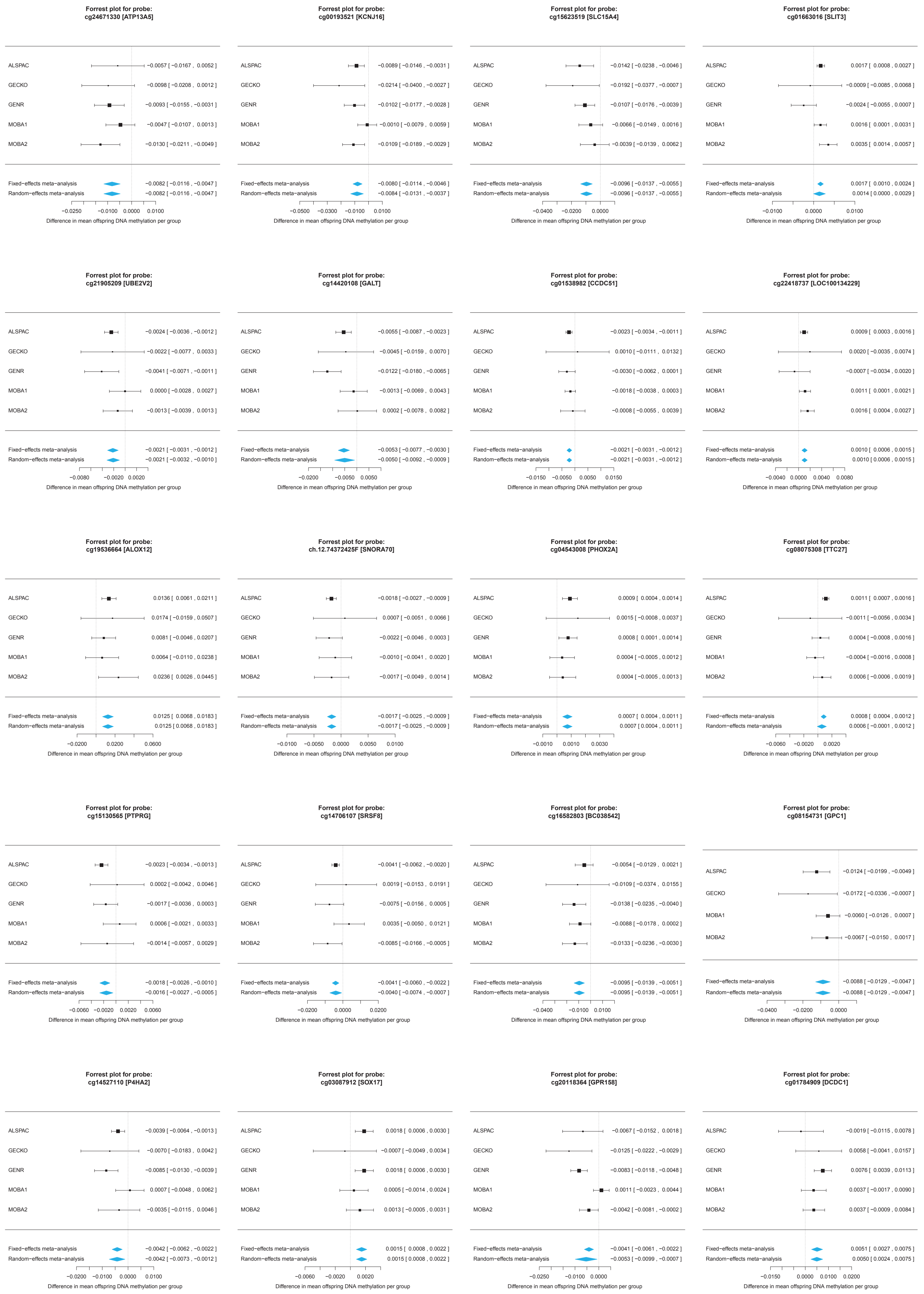

Forrest plot for probe: cg24671330 [ATP13A5]
ALSPAC −0.0057 [−0.0167 , 0.0052]
GECKO −0.0098 [−0.0208 , 0.0012]
GENR −0.0093 [−0.0155 , −0.0031]
MOBA1 −0.0047 [−0.0107 , 0.0013]
MOBA2 −0.0130 [−0.0211 , −0.0049]
Fixed−effects meta−analysis −0.0082 [−0.0116 , −0.0047]
Random−effects meta−analysis −0.0082 [−0.0116 , −0.0047]
−0.0250 −0.0100 0.0000 0.0100
Difference in mean offspring DNA methylation per group
Forrest plot for probe: cg00193521 [KCNJ16]
ALSPAC −0.0089 [−0.0146 , −0.0031]
GECKO −0.0214 [−0.0400 , −0.0027]
GENR −0.0102 [−0.0177 , −0.0028]
MOBA1 −0.0010 [−0.0079 , 0.0059]
MOBA2 −0.0109 [−0.0189 , −0.0029]
Fixed−effects meta−analysis −0.0080 [−0.0114 , −0.0046]
Random−effects meta−analysis −0.0084 [−0.0131 , −0.0037]
−0.0500 −0.0300 −0.0100 0.0100
Difference in mean offspring DNA methylation per group
Forrest plot for probe: cg15623519 [SLC15A4]
ALSPAC −0.0142 [−0.0238 , −0.0046]
GECKO −0.0192 [−0.0377 , −0.0007]
GENR −0.0107 [−0.0176 , −0.0039]
MOBA1 −0.0066 [−0.0149 , 0.0016]
MOBA2 −0.0039 [−0.0139 , 0.0062]
Fixed−effects meta−analysis −0.0096 [−0.0137 , −0.0055]
Random−effects meta−analysis −0.0096 [−0.0137 , −0.0055]
−0.0400 −0.0200 0.0000
Difference in mean offspring DNA methylation per group
Forrest plot for probe: cg01663016 [SLIT3]
ALSPAC 0.0017 [0.0008 , 0.0027]
GECKO −0.0009 [−0.0085 , 0.0068]
GENR −0.0024 [−0.0055 , 0.0007]
MOBA1 0.0016 [0.0001 , 0.0031]
MOBA2 0.0035 [0.0014 , 0.0057]
Fixed−effects meta−analysis 0.0017 [0.0010 , 0.0024]
Random−effects meta−analysis 0.0014 [0.0000 , 0.0029]
−0.0100 0.0000 0.0100
Difference in mean offspring DNA methylation per group
Forrest plot for probe: cg21905209 [UBE2V2]
ALSPAC −0.0024 [−0.0036 , −0.0012]
GECKO −0.0022 [−0.0077 , 0.0033]
GENR −0.0041 [−0.0071 , −0.0011]
MOBA1 0.0000 [−0.0028 , 0.0027]
MOBA2 −0.0013 [−0.0039 , 0.0013]
Fixed−effects meta−analysis −0.0021 [−0.0031 , −0.0012]
Random−effects meta−analysis −0.0021 [−0.0032 , −0.0010]
−0.0080 −0.0020 0.0020
Difference in mean offspring DNA methylation per group
Forrest plot for probe: cg14420108 [GALT]
ALSPAC −0.0055 [−0.0087 , −0.0023]
GECKO −0.0045 [−0.0159 , 0.0070]
GENR −0.0122 [−0.0180 , −0.0065]
MOBA1 −0.0013 [−0.0069 , 0.0043]
MOBA2 0.0002 [−0.0078 , 0.0082]
Fixed−effects meta−analysis −0.0053 [−0.0077 , −0.0030]
Random−effects meta−analysis −0.0050 [−0.0092 , −0.0009]
−0.0200 −0.0050 0.0050
Difference in mean offspring DNA methylation per group
Forrest plot for probe: cg01538982 [CCDC51]
ALSPAC −0.0023 [−0.0034 , −0.0011]
GECKO 0.0010 [−0.0111 , 0.0132]
GENR −0.0030 [−0.0062 , 0.0001]
MOBA1 −0.0018 [−0.0038 , 0.0003]
MOBA2 −0.0008 [−0.0055 , 0.0039]
Fixed−effects meta−analysis −0.0021 [−0.0031 , −0.0012]
Random−effects meta−analysis −0.0021 [−0.0031 , −0.0012]
−0.0150 −0.0050 0.0050 0.0150
Difference in mean offspring DNA methylation per group
Forrest plot for probe: cg22418737 [LOC100134229]
ALSPAC 0.0009 [0.0003 , 0.0016]
GECKO 0.0020 [−0.0035 , 0.0074]
GENR −0.0007 [−0.0034 , 0.0020]
MOBA1 0.0011 [0.0001 , 0.0021]
MOBA2 0.0016 [0.0004 , 0.0027]
Fixed−effects meta−analysis 0.0010 [0.0006 , 0.0015]
Random−effects meta−analysis 0.0010 [0.0006 , 0.0015]
−0.0040 0.0000 0.0040 0.0080
Difference in mean offspring DNA methylation per group
Forrest plot for probe: cg19536664 [ALOX12]
ALSPAC 0.0136 [0.0061 , 0.0211]
GECKO 0.0174 [−0.0159 , 0.0507]
GENR 0.0081 [−0.0046 , 0.0207]
MOBA1 0.0064 [−0.0110 , 0.0238]
MOBA2 0.0236 [0.0026 , 0.0445]
Fixed−effects meta−analysis 0.0125 [0.0068 , 0.0183]
Random−effects meta−analysis 0.0125 [0.0068 , 0.0183]
−0.0200 0.0200 0.0600
Difference in mean offspring DNA methylation per group
Forrest plot for probe: ch.12.74372425F [SNORA70]
ALSPAC −0.0018 [−0.0027 , −0.0009]
GECKO 0.0007 [−0.0051 , 0.0066]
GENR −0.0022 [−0.0046 , 0.0003]
MOBA1 −0.0010 [−0.0041 , 0.0020]
MOBA2 −0.0017 [−0.0049 , 0.0014]
Fixed−effects meta−analysis −0.0017 [−0.0025 , −0.0009]
Random−effects meta−analysis −0.0017 [−0.0025 , −0.0009]
−0.0100 −0.0050 0.0000 0.0050 0.0100
Difference in mean offspring DNA methylation per group
Forrest plot for probe: cg04543008 [PHOX2A]
ALSPAC 0.0009 [0.0004 , 0.0014]
GECKO 0.0015 [−0.0008 , 0.0037]
GENR 0.0008 [0.0001 , 0.0014]
MOBA1 0.0004 [−0.0005 , 0.0012]
MOBA2 0.0004 [−0.0005 , 0.0013]
Fixed−effects meta−analysis 0.0007 [0.0004 , 0.0011]
Random−effects meta−analysis 0.0007 [0.0004 , 0.0011]
−0.0010 0.0010 0.0030
Difference in mean offspring DNA methylation per group
Forrest plot for probe: cg08075308 [TTC27]
ALSPAC 0.0011 [0.0007 , 0.0016]
GECKO −0.0011 [−0.0056 , 0.0034]
GENR 0.0004 [−0.0008 , 0.0016]
MOBA1 −0.0004 [−0.0016 , 0.0008]
MOBA2 0.0006 [−0.0006 , 0.0019]
Fixed−effects meta−analysis 0.0008 [0.0004 , 0.0012]
Random−effects meta−analysis 0.0006 [−0.0001 , 0.0012]
−0.0060 −0.0020 0.0020
Difference in mean offspring DNA methylation per group
Forrest plot for probe: cg15130565 [PTPRG]
ALSPAC −0.0023 [−0.0034 , −0.0013]
GECKO 0.0002 [−0.0042 , 0.0046]
GENR −0.0017 [−0.0036 , 0.0003]
MOBA1 0.0006 [−0.0021 , 0.0033]
MOBA2 −0.0014 [−0.0057 , 0.0029]
Fixed−effects meta−analysis −0.0018 [−0.0026 , −0.0010]
Random−effects meta−analysis −0.0016 [−0.0027 , −0.0005]
−0.0060 −0.0020 0.0020 0.0060
Difference in mean offspring DNA methylation per group
Forrest plot for probe: cg14706107 [SRSF8]
ALSPAC −0.0041 [−0.0062 , −0.0020]
GECKO 0.0019 [−0.0153 , 0.0191]
GENR −0.0075 [−0.0156 , 0.0005]
MOBA1 0.0035 [−0.0050 , 0.0121]
MOBA2 −0.0085 [−0.0166 , −0.0005]
Fixed−effects meta−analysis −0.0041 [−0.0060 , −0.0022]
Random−effects meta−analysis −0.0040 [−0.0074 , −0.0007]
−0.0200 0.0000 0.0200
Difference in mean offspring DNA methylation per group
Forrest plot for probe: cg16582803 [BC038542]
ALSPAC −0.0054 [−0.0129 , 0.0021]
GECKO −0.0109 [−0.0374 , 0.0155]
GENR −0.0138 [−0.0235 , −0.0040]
MOBA1 −0.0088 [−0.0178 , 0.0002]
MOBA2 −0.0133 [−0.0236 , −0.0030]
Fixed−effects meta−analysis −0.0095 [−0.0139 , −0.0051]
Random−effects meta−analysis −0.0095 [−0.0139 , −0.0051]
−0.0400 −0.0100 0.0100
Difference in mean offspring DNA methylation per group
Forrest plot for probe: cg08154731 [GPC1]
ALSPAC −0.0124 [−0.0199 , −0.0049]
GECKO −0.0172 [−0.0336 , −0.0007]
MOBA1 −0.0060 [−0.0126 , 0.0007]
MOBA2 −0.0067 [−0.0150 , 0.0017]
Fixed−effects meta−analysis −0.0088 [−0.0129 , −0.0047]
Random−effects meta−analysis −0.0088 [−0.0129 , −0.0047]
−0.0400 −0.0200 0.0000
Difference in mean offspring DNA methylation per group
Forrest plot for probe: cg14527110 [P4HA2]
ALSPAC −0.0039 [−0.0064 , −0.0013]
GECKO −0.0070 [−0.0183 , 0.0042]
GENR −0.0085 [−0.0130 , −0.0039]
MOBA1 0.0007 [−0.0048 , 0.0062]
MOBA2 −0.0035 [−0.0115 , 0.0046]
Fixed−effects meta−analysis −0.0042 [−0.0062 , −0.0022]
Random−effects meta−analysis −0.0042 [−0.0073 , −0.0012]
−0.0200 −0.0100 0.0000 0.0100
Difference in mean offspring DNA methylation per group
Forrest plot for probe: cg03087912 [SOX17]
ALSPAC 0.0018 [0.0006 , 0.0030]
GECKO −0.0007 [−0.0049 , 0.0034]
GENR 0.0018 [0.0006 , 0.0030]
MOBA1 0.0005 [−0.0014 , 0.0024]
MOBA2 0.0013 [−0.0005 , 0.0031]
Fixed−effects meta−analysis 0.0015 [0.0008 , 0.0022]
Random−effects meta−analysis 0.0015 [0.0008 , 0.0022]
−0.0060 −0.0020 0.0020
Difference in mean offspring DNA methylation per group
Forrest plot for probe: cg20118364 [GPR158]
ALSPAC −0.0067 [−0.0152 , 0.0018]
GECKO −0.0125 [−0.0222 , −0.0029]
GENR −0.0083 [−0.0118 , −0.0048]
MOBA1 0.0011 [−0.0023 , 0.0044]
MOBA2 −0.0042 [−0.0081 , −0.0002]
Fixed−effects meta−analysis −0.0041 [−0.0061 , −0.0022]
Random−effects meta−analysis −0.0053 [−0.0099 , −0.0007]
−0.0250 −0.0100 0.0000
Difference in mean offspring DNA methylation per group
Forrest plot for probe: cg01784909 [DCDC1]
ALSPAC −0.0019 [−0.0115 , 0.0078]
GECKO 0.0058 [−0.0041 , 0.0157]
GENR 0.0076 [0.0039 , 0.0113]
MOBA1 0.0037 [−0.0017 , 0.0090]
MOBA2 0.0037 [−0.0009 , 0.0084]
Fixed−effects meta−analysis 0.0051 [0.0027 , 0.0075]
Random−effects meta−analysis 0.0050 [0.0024 , 0.0075]
−0.0150 0.0000 0.0100 0.0200
Difference in mean offspring DNA methylation per group

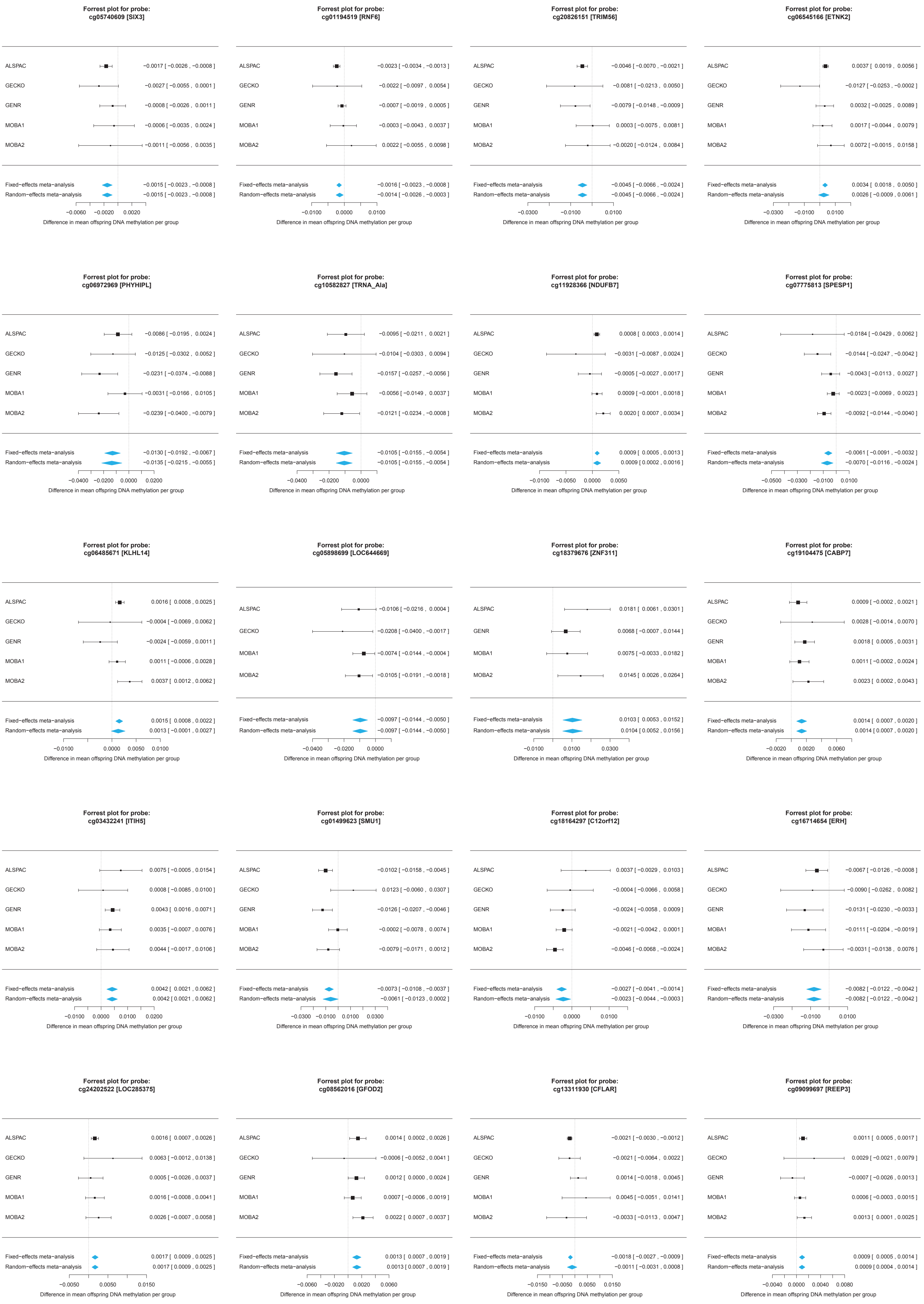
Forrest plot for probe:
cg05740609 [SIX3]
ALSPAC −0.0017 [−0.0026 , −0.0008]
GECKO −0.0027 [−0.0055 , 0.0001]
GENR −0.0008 [−0.0026 , 0.0011]
MOBA1 −0.0006 [−0.0035 , 0.0024]
MOBA2 −0.0011 [−0.0056 , 0.0035]
Fixed−effects meta−analysis −0.0015 [−0.0023 , −0.0008]
Random−effects meta−analysis −0.0015 [−0.0023 , −0.0008]
−0.0060 −0.0020 0.0020
Difference in mean offspring DNA methylation per group
Forrest plot for probe:
cg01194519 [RNF6]
ALSPAC −0.0023 [−0.0034 , −0.0013]
GECKO −0.0022 [−0.0097 , 0.0054]
GENR −0.0007 [−0.0019 , 0.0005]
MOBA1 −0.0003 [−0.0043 , 0.0037]
MOBA2 0.0022 [−0.0055 , 0.0098]
Fixed−effects meta−analysis −0.0016 [−0.0023 , −0.0008]
Random−effects meta−analysis −0.0014 [−0.0026 , −0.0003]
−0.0100 0.0000 0.0100
Difference in mean offspring DNA methylation per group
Forrest plot for probe:
cg20826151 [TRIM56]
ALSPAC −0.0046 [−0.0070 , −0.0021]
GECKO −0.0081 [−0.0213 , 0.0050]
GENR −0.0079 [−0.0148 , −0.0009]
MOBA1 0.0003 [−0.0075 , 0.0081]
MOBA2 −0.0020 [−0.0124 , 0.0084]
Fixed−effects meta−analysis −0.0045 [−0.0066 , −0.0024]
Random−effects meta−analysis −0.0045 [−0.0066 , −0.0024]
−0.0300 −0.0100 0.0100
Difference in mean offspring DNA methylation per group
Forrest plot for probe:
cg06545166 [ETNK2]
ALSPAC 0.0037 [0.0019 , 0.0056]
GECKO −0.0127 [−0.0253 , −0.0002]
GENR 0.0032 [−0.0025 , 0.0089]
MOBA1 0.0017 [−0.0044 , 0.0079]
MOBA2 0.0072 [−0.0015 , 0.0158]
Fixed−effects meta−analysis 0.0034 [0.0018 , 0.0050]
Random−effects meta−analysis 0.0026 [−0.0009 , 0.0061]
−0.0300 −0.0100 0.0100
Difference in mean offspring DNA methylation per group
Forrest plot for probe:
cg06972969 [PHYHIPL]
ALSPAC −0.0086 [−0.0195 , 0.0024]
GECKO −0.0125 [−0.0302 , 0.0052]
GENR −0.0231 [−0.0374 , −0.0088]
MOBA1 −0.0031 [−0.0166 , 0.0105]
MOBA2 −0.0239 [−0.0400 , −0.0079]
Fixed−effects meta−analysis −0.0130 [−0.0192 , −0.0067]
Random−effects meta−analysis −0.0135 [−0.0215 , −0.0055]
−0.0400 −0.0200 0.0000 0.0200
Difference in mean offspring DNA methylation per group
Forrest plot for probe:
cg10582827 [TRNA_Ala]
ALSPAC −0.0095 [−0.0211 , 0.0021]
GECKO −0.0104 [−0.0303 , 0.0094]
GENR −0.0157 [−0.0257 , −0.0056]
MOBA1 −0.0056 [−0.0149 , 0.0037]
MOBA2 −0.0121 [−0.0234 , −0.0008]
Fixed−effects meta−analysis −0.0105 [−0.0155 , −0.0054]
Random−effects meta−analysis −0.0105 [−0.0155 , −0.0054]
−0.0400 −0.0200 0.0000
Difference in mean offspring DNA methylation per group
Forrest plot for probe:
cg11928366 [NDUFB7]
ALSPAC 0.0008 [0.0003 , 0.0014]
GECKO −0.0031 [−0.0087 , 0.0024]
GENR −0.0005 [−0.0027 , 0.0017]
MOBA1 0.0009 [−0.0001 , 0.0018]
MOBA2 0.0020 [0.0007 , 0.0034]
Fixed−effects meta−analysis 0.0009 [0.0005 , 0.0013]
Random−effects meta−analysis 0.0009 [0.0002 , 0.0016]
−0.0100 −0.0050 0.0000 0.0050
Difference in mean offspring DNA methylation per group
Forrest plot for probe:
cg07775813 [SPESP1]
ALSPAC −0.0184 [−0.0429 , 0.0062]
GECKO −0.0144 [−0.0247 , −0.0042]
GENR −0.0043 [−0.0113 , 0.0027]
MOBA1 −0.0023 [−0.0069 , 0.0023]
MOBA2 −0.0092 [−0.0144 , −0.0040]
Fixed−effects meta−analysis −0.0061 [−0.0091 , −0.0032]
Random−effects meta−analysis −0.0070 [−0.0116 , −0.0024]
−0.0500 −0.0300 −0.0100 0.0100
Difference in mean offspring DNA methylation per group
Forrest plot for probe:
cg06485671 [KLHL14]
ALSPAC 0.0016 [0.0008 , 0.0025]
GECKO −0.0004 [−0.0069 , 0.0062]
GENR −0.0024 [−0.0059 , 0.0011]
MOBA1 0.0011 [−0.0006 , 0.0028]
MOBA2 0.0037 [0.0012 , 0.0062]
Fixed−effects meta−analysis 0.0015 [0.0008 , 0.0022]
Random−effects meta−analysis 0.0013 [−0.0001 , 0.0027]
−0.0100 0.0000 0.0050 0.0100
Difference in mean offspring DNA methylation per group
Forrest plot for probe:
cg05898699 [LOC644669]
ALSPAC −0.0106 [−0.0216 , 0.0004]
GECKO −0.0208 [−0.0400 , −0.0017]
MOBA1 −0.0074 [−0.0144 , −0.0004]
MOBA2 −0.0105 [−0.0191 , −0.0018]
Fixed−effects meta−analysis −0.0097 [−0.0144 , −0.0050]
Random−effects meta−analysis −0.0097 [−0.0144 , −0.0050]
−0.0400 −0.0200 0.0000
Difference in mean offspring DNA methylation per group
Forrest plot for probe:
cg18379676 [ZNF311]
ALSPAC 0.0181 [0.0061 , 0.0301]
GENR 0.0068 [−0.0007 , 0.0144]
MOBA1 0.0075 [−0.0033 , 0.0182]
MOBA2 0.0145 [0.0026 , 0.0264]
Fixed−effects meta−analysis 0.0103 [0.0053 , 0.0152]
Random−effects meta−analysis 0.0104 [0.0052 , 0.0156]
−0.0100 0.0100 0.0300
Difference in mean offspring DNA methylation per group
Forrest plot for probe:
cg19104475 [CABP7]
ALSPAC 0.0009 [−0.0002 , 0.0021]
GECKO 0.0028 [−0.0014 , 0.0070]
GENR 0.0018 [0.0005 , 0.0031]
MOBA1 0.0011 [−0.0002 , 0.0024]
MOBA2 0.0023 [0.0002 , 0.0043]
Fixed−effects meta−analysis 0.0014 [0.0007 , 0.0020]
Random−effects meta−analysis 0.0014 [0.0007 , 0.0020]
−0.0020 0.0020 0.0060
Difference in mean offspring DNA methylation per group
Forrest plot for probe:
cg03432241 [ITIH5]
ALSPAC 0.0075 [−0.0005 , 0.0154]
GECKO 0.0008 [−0.0085 , 0.0100]
GENR 0.0043 [0.0016 , 0.0071]
MOBA1 0.0035 [−0.0007 , 0.0076]
MOBA2 0.0044 [−0.0017 , 0.0106]
Fixed−effects meta−analysis 0.0042 [0.0021 , 0.0062]
Random−effects meta−analysis 0.0042 [0.0021 , 0.0062]
−0.0100 0.0000 0.0100 0.0200
Difference in mean offspring DNA methylation per group
Forrest plot for probe:
cg01499623 [SMU1]
ALSPAC −0.0102 [−0.0158 , −0.0045]
GECKO 0.0123 [−0.0060 , 0.0307]
GENR −0.0126 [−0.0207 , −0.0046]
MOBA1 −0.0002 [−0.0078 , 0.0074]
MOBA2 −0.0079 [−0.0171 , 0.0012]
Fixed−effects meta−analysis −0.0073 [−0.0108 , −0.0037]
Random−effects meta−analysis −0.0061 [−0.0123 , 0.0002]
−0.0300 −0.0100 0.0100 0.0300
Difference in mean offspring DNA methylation per group
Forrest plot for probe:
cg18164297 [C12orf12]
ALSPAC 0.0037 [−0.0029 , 0.0103]
GECKO −0.0004 [−0.0066 , 0.0058]
GENR −0.0024 [−0.0058 , 0.0009]
MOBA1 −0.0021 [−0.0042 , 0.0001]
MOBA2 −0.0046 [−0.0068 , −0.0024]
Fixed−effects meta−analysis −0.0027 [−0.0041 , −0.0014]
Random−effects meta−analysis −0.0023 [−0.0044 , −0.0003]
−0.0100 0.0000 0.0100
Difference in mean offspring DNA methylation per group
Forrest plot for probe:
cg16714654 [ERH]
ALSPAC −0.0067 [−0.0126 , −0.0008]
GECKO −0.0090 [−0.0262 , 0.0082]
GENR −0.0131 [−0.0230 , −0.0033]
MOBA1 −0.0111 [−0.0204 , −0.0019]
MOBA2 −0.0031 [−0.0138 , 0.0076]
Fixed−effects meta−analysis −0.0082 [−0.0122 , −0.0042]
Random−effects meta−analysis −0.0082 [−0.0122 , −0.0042]
−0.0300 −0.0100 0.0100
Difference in mean offspring DNA methylation per group
Forrest plot for probe:
cg24202522 [LOC285375]
ALSPAC 0.0016 [0.0007 , 0.0026]
GECKO 0.0063 [−0.0012 , 0.0138]
GENR 0.0005 [−0.0026 , 0.0037]
MOBA1 0.0016 [−0.0008 , 0.0041]
MOBA2 0.0026 [−0.0007 , 0.0058]
Fixed−effects meta−analysis 0.0017 [0.0009 , 0.0025]
Random−effects meta−analysis 0.0017 [0.0009 , 0.0025]
−0.0050 0.0050 0.0150
Difference in mean offspring DNA methylation per group
Forrest plot for probe:
cg08562016 [GFOD2]
ALSPAC 0.0014 [0.0002 , 0.0026]
GECKO −0.0006 [−0.0052 , 0.0041]
GENR 0.0012 [0.0000 , 0.0024]
MOBA1 0.0007 [−0.0006 , 0.0019]
MOBA2 0.0022 [0.0007 , 0.0037]
Fixed−effects meta−analysis 0.0013 [0.0007 , 0.0019]
Random−effects meta−analysis 0.0013 [0.0007 , 0.0019]
−0.0060 −0.0020 0.0020 0.0060
Difference in mean offspring DNA methylation per group
Forrest plot for probe:
cg13311930 [CFLAR]
ALSPAC −0.0021 [−0.0030 , −0.0012]
GECKO −0.0021 [−0.0064 , 0.0022]
GENR 0.0014 [−0.0018 , 0.0045]
MOBA1 0.0045 [−0.0051 , 0.0141]
MOBA2 −0.0033 [−0.0113 , 0.0047]
Fixed−effects meta−analysis −0.0018 [−0.0027 , −0.0009]
Random−effects meta−analysis −0.0011 [−0.0031 , 0.0008]
−0.0150 −0.0050 0.0050 0.0150
Difference in mean offspring DNA methylation per group
Forrest plot for probe:
cg09099697 [REEP3]
ALSPAC 0.0011 [0.0005 , 0.0017]
GECKO 0.0029 [−0.0021 , 0.0079]
GENR −0.0007 [−0.0026 , 0.0013]
MOBA1 0.0006 [−0.0003 , 0.0015]
MOBA2 0.0013 [0.0001 , 0.0025]
Fixed−effects meta−analysis 0.0009 [0.0005 , 0.0014]
Random−effects meta−analysis 0.0009 [0.0004 , 0.0014]
−0.0040 0.0000 0.0040 0.0080
Difference in mean offspring DNA methylation per group

Forrest plot for probe: cg19595170 [SRF]

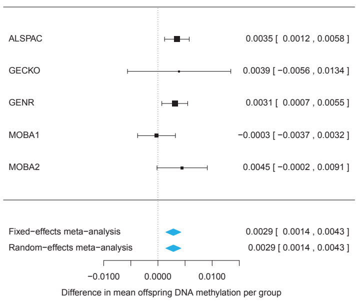

Forrest plot for probe: cg11879776 [CST8]

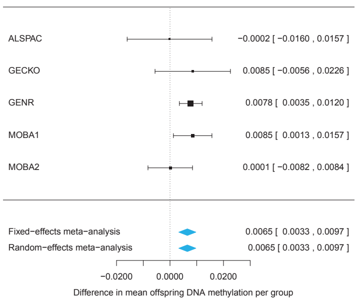

Forrest plot for probe: cg27455331 [FLJ40194]

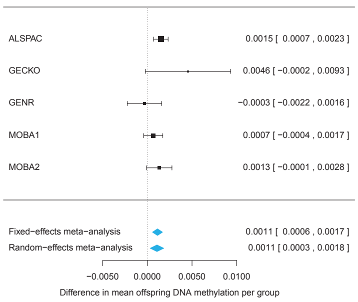

Forrest plot for probe: cg21545857 [D2HGDH]

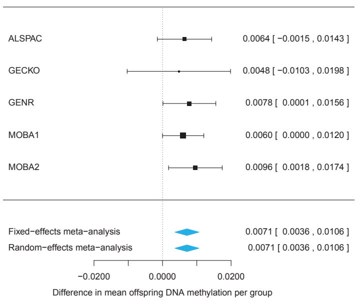

Forrest plot for probe: cg23915527 [TRNA_Val]

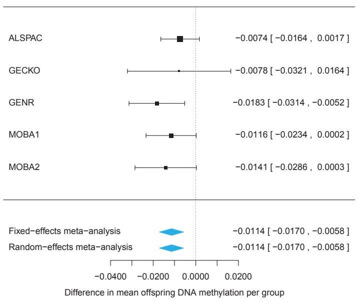

Forrest plot for probe: cg08484934 [C15orf59]

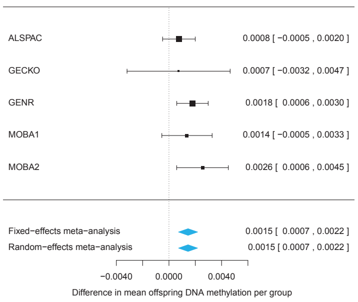

Forrest plot for probe: cg18980733 [GFRA3]

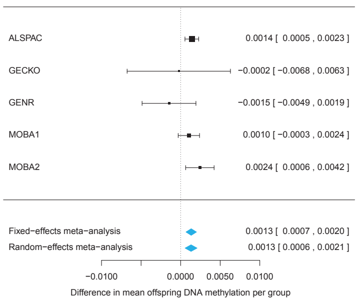

Forrest plot for probe: cg15680902 [IL1RAP]

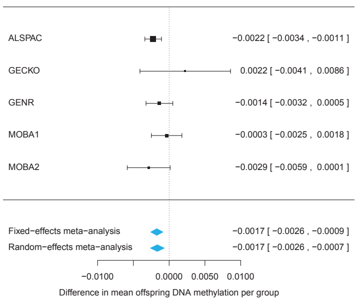

Forrest plot for probe: cg12662931 [DUSP19]

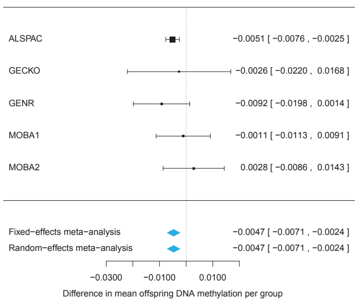

Forrest plot for probe: cg19914751 [QARS]

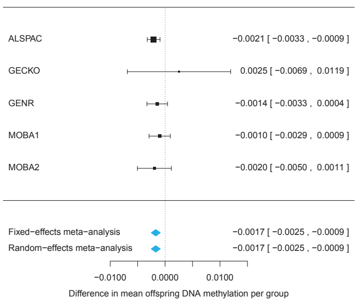

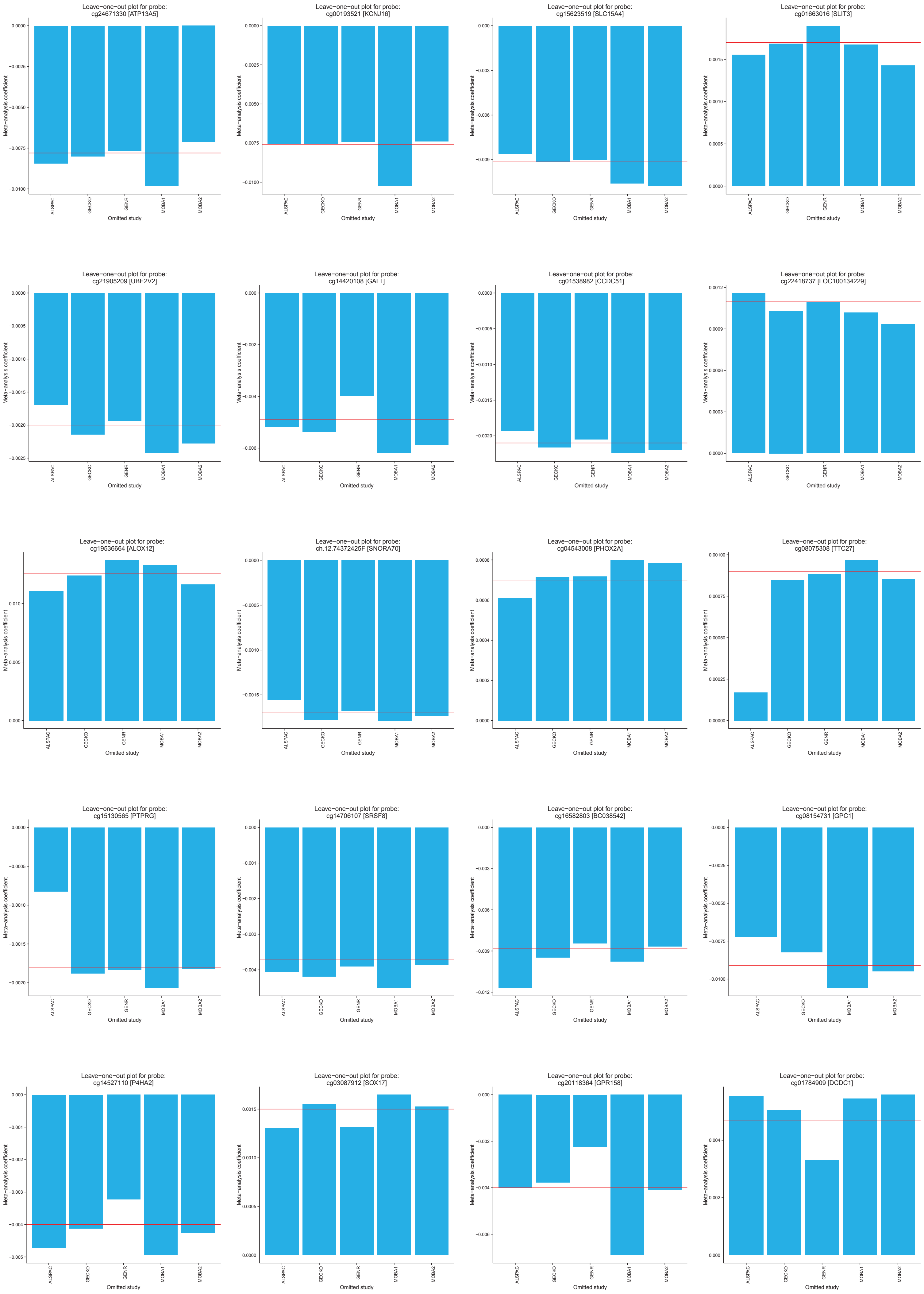
Leave−one−out plot for probe: cg24671330 [ATP13A5]
Leave−one−out plot for probe: cg00193521 [KCNJ16]
Leave−one−out plot for probe: cg15623519 [SLC15A4]
Leave−one−out plot for probe: cg01663016 [SLIT3]
Leave−one−out plot for probe: cg21905209 [UBE2V2]
Leave−one−out plot for probe: cg14420108 [GALT]
Leave−one−out plot for probe: cg01538982 [CCDC51]
Leave−one−out plot for probe: cg22418737 [LOC100134229]
Leave−one−out plot for probe: cg19536664 [ALOX12]
Leave−one−out plot for probe: ch.12.74372425F [SNORA70]
Leave−one−out plot for probe: cg04543008 [PHOX2A]
Leave−one−out plot for probe: cg08075308 [TTC27]
Leave−one−out plot for probe: cg15130565 [PTPRG]
Leave−one−out plot for probe: cg14706107 [SRSF8]
Leave−one−out plot for probe: cg16582803 [BC038542]
Leave−one−out plot for probe: cg08154731 [GPC1]
Leave−one−out plot for probe: cg14527110 [P4HA2]
Leave−one−out plot for probe: cg03087912 [SOX17]
Leave−one−out plot for probe: cg20118364 [GPR158]
Leave−one−out plot for probe: cg01784909 [DCDC1]
Meta−analysis coefficient
Omitted study
ALSPAC
GECKO
GENR
MOBA1
MOBA2

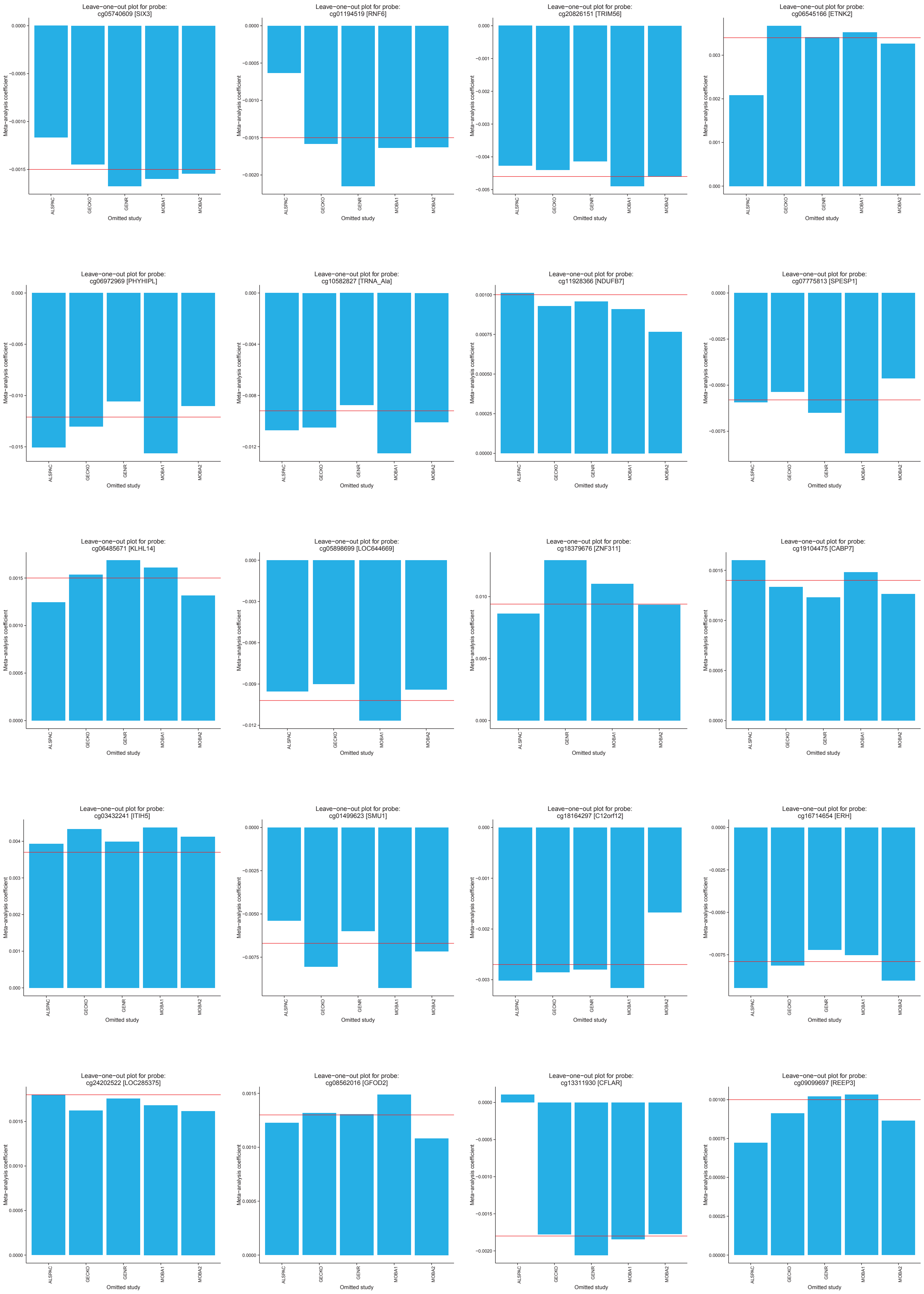

Leave−one−out plot for probe:
cg05740609 [SIX3]
Meta−analysis coefficient
0.0000
−0.0005
−0.0010
−0.0015
ALSPAC
GECKO
GENR
MOBA1
MOBA2
Omitted study
Leave−one−out plot for probe:
cg01194519 [RNF6]
Meta−analysis coefficient
0.0000
−0.0005
−0.0010
−0.0015
−0.0020
ALSPAC
GECKO
GENR
MOBA1
MOBA2
Omitted study
Leave−one−out plot for probe:
cg20826151 [TRIM56]
Meta−analysis coefficient
0.000
−0.001
−0.002
−0.003
−0.004
−0.005
ALSPAC
GECKO
GENR
MOBA1
MOBA2
Omitted study
Leave−one−out plot for probe:
cg06545166 [ETNK2]
Meta−analysis coefficient
0.003
0.002
0.001
0.000
ALSPAC
GECKO
GENR
MOBA1
MOBA2
Omitted study
Leave−one−out plot for probe:
cg06972969 [PHYHIPL]
Meta−analysis coefficient
0.000
−0.005
−0.010
−0.015
ALSPAC
GECKO
GENR
MOBA1
MOBA2
Omitted study
Leave−one−out plot for probe:
cg10582827 [TRNA_Ala]
Meta−analysis coefficient
0.000
−0.004
−0.008
−0.012
ALSPAC
GECKO
GENR
MOBA1
MOBA2
Omitted study
Leave−one−out plot for probe:
cg11928366 [NDUFB7]
Meta−analysis coefficient
0.00100
0.00075
0.00050
0.00025
0.00000
ALSPAC
GECKO
GENR
MOBA1
MOBA2
Omitted study
Leave−one−out plot for probe:
cg07775813 [SPESP1]
Meta−analysis coefficient
0.0000
−0.0025
−0.0050
−0.0075
ALSPAC
GECKO
GENR
MOBA1
MOBA2
Omitted study
Leave−one−out plot for probe:
cg06485671 [KLHL14]
Meta−analysis coefficient
0.0015
0.0010
0.0005
0.0000
ALSPAC
GECKO
GENR
MOBA1
MOBA2
Omitted study
Leave−one−out plot for probe:
cg05898699 [LOC644669]
Meta−analysis coefficient
0.000
−0.003
−0.006
−0.009
−0.012
ALSPAC
GECKO
MOBA1
MOBA2
Omitted study
Leave−one−out plot for probe:
cg18379676 [ZNF311]
Meta−analysis coefficient
0.010
0.005
0.000
ALSPAC
GENR
MOBA1
MOBA2
Omitted study
Leave−one−out plot for probe:
cg19104475 [CABP7]
Meta−analysis coefficient
0.0015
0.0010
0.0005
0.0000
ALSPAC
GECKO
GENR
MOBA1
MOBA2
Omitted study
Leave−one−out plot for probe:
cg03432241 [ITIH5]
Meta−analysis coefficient
0.004
0.003
0.002
0.001
0.000
ALSPAC
GECKO
GENR
MOBA1
MOBA2
Omitted study
Leave−one−out plot for probe:
cg01499623 [SMU1]
Meta−analysis coefficient
0.0000
−0.0025
−0.0050
−0.0075
ALSPAC
GECKO
GENR
MOBA1
MOBA2
Omitted study
Leave−one−out plot for probe:
cg18164297 [C12orf12]
Meta−analysis coefficient
0.000
−0.001
−0.002
−0.003
ALSPAC
GECKO
GENR
MOBA1
MOBA2
Omitted study
Leave−one−out plot for probe:
cg16714654 [ERH]
Meta−analysis coefficient
0.0000
−0.0025
−0.0050
−0.0075
ALSPAC
GECKO
GENR
MOBA1
MOBA2
Omitted study
Leave−one−out plot for probe:
cg24202522 [LOC285375]
Meta−analysis coefficient
0.0015
0.0010
0.0005
0.0000
ALSPAC
GECKO
GENR
MOBA1
MOBA2
Omitted study
Leave−one−out plot for probe:
cg08562016 [GFOD2]
Meta−analysis coefficient
0.0015
0.0010
0.0005
0.0000
ALSPAC
GECKO
GENR
MOBA1
MOBA2
Omitted study
Leave−one−out plot for probe:
cg13311930 [CFLAR]
Meta−analysis coefficient
0.0000
−0.0005
−0.0010
−0.0015
−0.0020
ALSPAC
GECKO
GENR
MOBA1
MOBA2
Omitted study
Leave−one−out plot for probe:
cg09099697 [REEP3]
Meta−analysis coefficient
0.00100
0.00075
0.00050
0.00025
0.00000
ALSPAC
GECKO
GENR
MOBA1
MOBA2
Omitted study

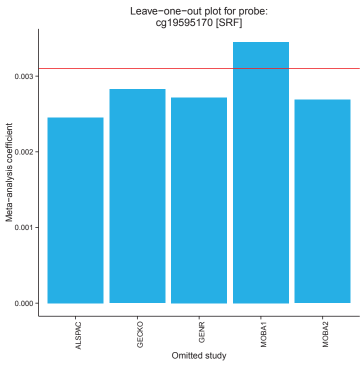
Leave−one−out plot for probe:
cg19595170 [SRF]
Meta−analysis coefficient
0.000
0.001
0.002
0.003
ALSPAC
GECKO
GENR
MOBA1
MOBA2
Omitted study

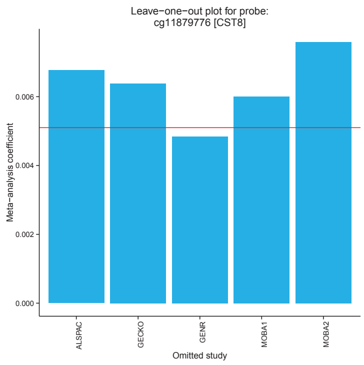
Leave−one−out plot for probe:
cg11879776 [CST8]
Meta−analysis coefficient
0.000
0.002
0.004
0.006
ALSPAC
GECKO
GENR
MOBA1
MOBA2
Omitted study

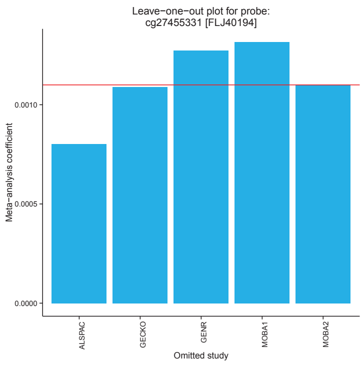
Leave−one−out plot for probe:
cg27455331 [FLJ40194]
Meta−analysis coefficient
0.0000
0.0005
0.0010
ALSPAC
GECKO
GENR
MOBA1
MOBA2
Omitted study

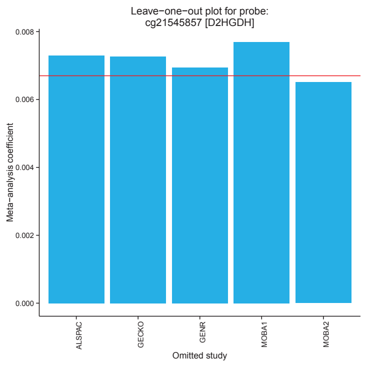
Leave−one−out plot for probe:
cg21545857 [D2HGDH]
Meta−analysis coefficient
0.000
0.002
0.004
0.006
0.008
ALSPAC
GECKO
GENR
MOBA1
MOBA2
Omitted study

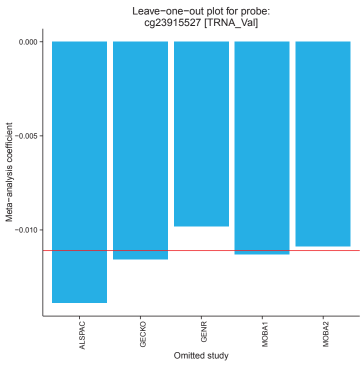
Leave−one−out plot for probe:
cg23915527 [TRNA_Val]
Meta−analysis coefficient
0.000
−0.005
−0.010
ALSPAC
GECKO
GENR
MOBA1
MOBA2
Omitted study

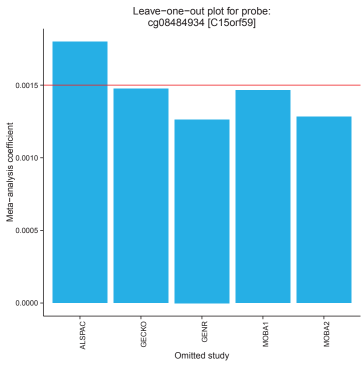
Leave−one−out plot for probe:
cg08484934 [C15orf59]
Meta−analysis coefficient
0.0000
0.0005
0.0010
0.0015
ALSPAC
GECKO
GENR
MOBA1
MOBA2
Omitted study

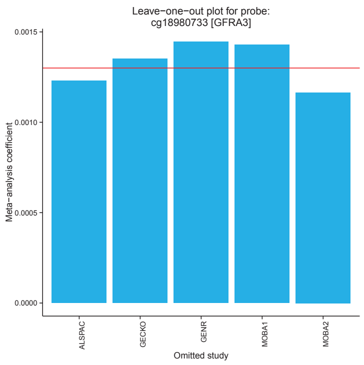
Leave−one−out plot for probe:
cg18980733 [GFRA3]
Meta−analysis coefficient
0.0000
0.0005
0.0010
0.0015
ALSPAC
GECKO
GENR
MOBA1
MOBA2
Omitted study

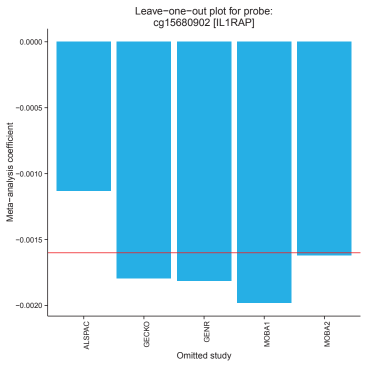
Leave−one−out plot for probe:
cg15680902 [IL1RAP]
Meta−analysis coefficient
0.0000
−0.0005
−0.0010
−0.0015
−0.0020
ALSPAC
GECKO
GENR
MOBA1
MOBA2
Omitted study

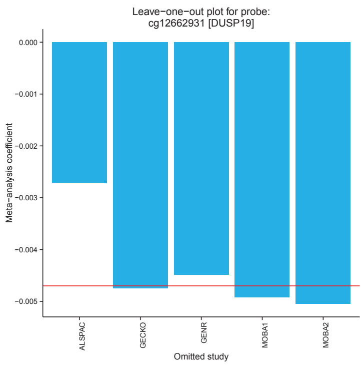
Leave−one−out plot for probe:
cg12662931 [DUSP19]
Meta−analysis coefficient
0.000
−0.001
−0.002
−0.003
−0.004
−0.005
ALSPAC
GECKO
GENR
MOBA1
MOBA2
Omitted study

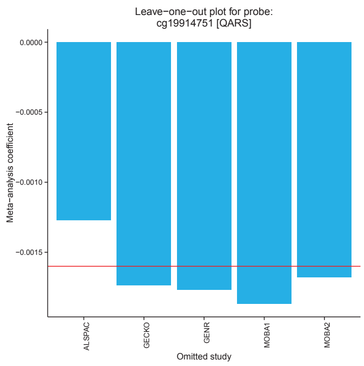
Leave−one−out plot for probe:
cg19914751 [QARS]
Meta−analysis coefficient
0.0000
−0.0005
−0.0010
−0.0015
ALSPAC
GECKO
GENR
MOBA1
MOBA2
Omitted study
